# Editorial: Single-cell OMICs analyses in cardiovascular diseases

**DOI:** 10.3389/fcvm.2024.1413184

**Published:** 2024-05-06

**Authors:** Abhijeet Rajendra Sonawane, Michel Pucéat, Hanjoong Jo

**Affiliations:** ^1^ Center for Interdisciplinary Cardiovascular Sciences and Center for Excellences in Vascular Biology, Division of Cardiovascular Medicine, Department of Medicine, Brigham and Women’s Hospital, Harvard Medical School, Boston, MA, United States; ^2^ INSERM, Cardiovascular and Nutrition Center (C2VN), Aix-Marseille University, Marseille, France; ^3^ Wallace H. Coulter Department of Biomedical Engineering, Emory University and Georgia Institute of Technology, Atlanta, GA, United States

**Keywords:** cardiovascular diseases, single-cell omics, data analysis, atherosclerosis, heart valve disease (HVD)

**Editorial on the Research Topic** Single-cell OMICs analyses in cardiovascular diseases

Single-cell technologies have revolutionized the understanding of biological and pathological processes. They are now the driving force of cellular profiling across cardiovascular diseases (CVD) (1–5). They have enabled analysis of cellular composition with tissues and cell cultures, revealing the subtle nuances of heterogeneity among cell types and uncovering the dynamic changes in communication and signaling patterns triggered by disease.

Various omics modalities data can be generated now at single-cell resolution including joint and concomitant assays such as single cell RNA-sequencing (scRNA-seq) and single nucleus RNA-seq (snRNA-seq) for transcriptomics profiling, single cell assay for transposase-accessible chromatin with sequencing (scATAC-seq) for cell type specific profiling of epigenetic, and in turn, regulatory landscape, and cellular indexing of transcriptomes and epitopes-seq (CITE-seq) for profiling the surface proteins on different cell types. Recent advances, including spatial transcriptomics and single cell proteomics (6, 7) have allowed us to spatially resolve cell subtypes in active areas of disease tissues. These multiomics data types allow us to develop novel computational and bioinformatics methods to obtain a multidimensional view of the disease to uncover mechanisms, explain etiologies, and discover biomarkers. Single-cell data analysis tools such as Seurat (8, 9), and SingleCellExperiment (10) have democratized the capability of single cell analysis by beckoning researchers of all backgrounds to embark on a journey of exploration, regardless of their level of bioinformatics expertise. Applications such as SCHNAPPs have also enabled bench scientists to perform basic single cell analyses (11).

Understanding the impact of single cell research on CVD, including vascular and heart valve diseases, is crucial as it allows us to evaluate advancements in the field. Specific cells such as immune cells as well as cell-cell communication play a significant role in disease pathogenesis. Identifying unique cell types (cell subpopulations) and their behavior in the diseased tissue can elucidate their role in altering tissue structure and function.

In this special research topic, we intended to create a forum for current advances in single-cell techniques applied to cardiovascular disease areas. We invited articles on single cell technique and methods development, probing cellular heterogeneity, modern laboratory and in silico techniques, and its application to various vascular and valvular diseases. We received 18 submissions, out of which we selected 10 high quality peer reviewed articles for our collections. Below, we provide a brief overview of articles in this collection.

The systematic review conducted according to PRISMA standards from McQueen et al. provides us with extensive overview of the current state of single-cell research in cardiovascular medicine. Using proper exclusion and inclusion criterion, addressing the risk of bias analyses using tools, they provide a narrative synthesis of 34 articles (out of 791). They provide a comprehensive review of studies which include various single cell omics modalities such as scRNA-seq, scATAC-seq, CITE-seq, or combination thereof. They also include cell-type-specific discussions of the progress done in the research in cardiovascular diseases including, smooth muscle cells, macrophages, endothelial cells, and lymphocytes. Results from tissue and blood-specific analyses which included atherosclerotic lesions, cardiac and adventitial tissues, and blood, are also summarized to provide the reader important processes involved in atherosclerosis development and progression. Hu et al. review recent advances in scRNA-seq technology along with comparison of various gene amplification methods. They also discuss some of the standard workflow associated with single cell studies applied in CVD medicine. Su et al. present a focused review on role of diversity and abundance of immune cells such as macrophages, dendritic cells, and T cells, in cardiac homeostasis and effect of their infiltration on the diseased areas. They focus on immune heterogeneity in atherosclerosis, myocardial ischemia, and heart failure along with suggesting potentially new marker genes.

Li et al. continue the discussion on heart failure using the role of, relatively little explored, neutrophile extracellular traps (NET). Using conventional bioinformatic analysis on bulk and single cell RNA-seq datasets, they identify differentially expressed NET-related genes followed by neutrophile cell heterogeneity in heart failure and normal cardiac tissues followed by cellular differentiation and communication analyses identifying biomarkers associated with NETs in heart failure. Zhang et al. study myocardial infarction (MI) using public datasets and standard bioinformatics pipeline to identify IL1B and TLR2 as differentially expressed genes (DEGs) with most neighbors in protein-protein interaction network (PPI) in MI. While their findings provide valuable insights, there is a need for cautious interpretation due to the limitations inherent in the utilization of public datasets. Additionally, several seminal scRNA-seq studies highlighted the importance of sexual differences at the cell level in CVD. Marrero et al. study on sexual differences in peripheral artery disease (PAD) etiology utilizing scRNA-seq data. Mizrak et al. (12) using spatial transcriptomics uncovered male-specific smooth muscle cells subpopulation playing a key role in human thoracic aortic aneurysm. ScRNA-seq data from Shin et al. (13) allow to delineate the sex-difference in endothelial cells characteristics and function providing new clues about atherosclerotic diseases. These studies show that inclusion of biological sex in a proper experimental design (14) is of utmost importance to consider representativeness of the sample population and potential confounding variables.

Autoimmune conditions like cardiac and pulmonary sarcoidosis (CS/PS) are understudied yet potentially impactful on cardiovascular health. Daoud et al. conducted a systematic review using single-cell RNA-seq datasets to investigate PS and CS. They found increased immune cells and stromal populations in sarcoidosis tissues compared to controls, with sarcoidosis T cells and macrophages showing attenuated activation profiles. Abdominal aortic aneurysm (AAA) involves complex immune cell interactions and may exhibit autoimmune characteristics. Elster et al. studied the clonal expansion of T cells and B cells in AAA tissue using sc-RNA T cell receptor (TCR) and B cell receptor (BCR) sequencing using porcine pancreatic elastase mouse model. Wu et al. investigated macrophage regulation in AAA using scRNA-seq datasets from mouse models and humans. They identified IL-1B and THBS1 as co-upregulated genes across datasets, highlighting the complex immune involvement in AAA. Similarly, thoracic arch aneurysm (TAA), linked to bicuspid aortic valves (BAV), was investigated by Liu et al., revealing potential therapeutic genes. These studies enhance our grasp of aortic aneurysm pathophysiology, urging further investigation for improved cardiovascular disease management.

To summarize, the emergence of single-cell technologies has completely transformed our comprehension of cellular dynamics within cardiovascular disorders, notably in dissecting the intricate cellular compositions, diversities, and signaling modifications associated with diseases such as heart valve diseases and atherosclerosis. Through thorough assessments and leading-edge investigations, researchers have elucidated the presence and functions of diverse vascular and immune cell populations, indicated novel biomarkers, and underlined the molecular pathways influencing disease progression. These studies now complemented with spatial transcriptomics indicate the need of development of sophisticated bioinformatics tools for the integration of multiomics methodologies, which are needed to unravel the intricate complexities of cardiovascular pathophysiology, that will open avenues for targeted treatments and enhanced patient outcomes.
